# Relationship between fluctuations in glucose levels measured by continuous glucose monitoring and vascular endothelial dysfunction in type 2 diabetes mellitus

**DOI:** 10.1186/1475-2840-12-1

**Published:** 2013-01-02

**Authors:** Keiichi Torimoto, Yosuke Okada, Hiroko Mori, Yoshiya Tanaka

**Affiliations:** 1 First Department of Internal Medicine, School of Medicine, University of Occupational and Environmental Health, 1-1 Iseigaoka, Yahatanishi-ku, Kitakyushyu-shi, 807-8555, Japan

**Keywords:** Atherosclerosis, Type 2 diabetes mellitus, Endothelium, Glucose, Continuous glucose monitoring (CGM)

## Abstract

**Background:**

Fluctuations in blood glucose level cause endothelial dysfunction and play a critical role in onset and/or progression of atherosclerosis. We hypothesized that fluctuation in blood glucose levels correlate with vascular endothelial dysfunction and that this relationship can be assessed using common bedside medical devices.

**Methods:**

Fluctuations in blood glucose levels were measured over 24 hours by continuous glucose monitoring (CGM) on admission day 2 in 57 patients with type 2 diabetes mellitus. The reactive hyperemia index (RHI), an index of vascular endothelial function, was measured using peripheral arterial tonometry (EndoPAT) on admission day 3.

**Results:**

The natural logarithmic-scaled RHI (L_RHI) correlated with SD (r=−0.504; *P*<0.001), the mean amplitude of glycemic excursions (MAGE) (r=−0.571; *P*<0.001), mean postprandial glucose excursion (MPPGE) (r=−0.411; *P*=0.001) and percentage of time ≥200 mg/dl (r=−0.292; *P*=0.028). In 12 patients with hypoglycemia, L_RHI also correlated with the percentage of time at hypoglycemia (r=−0.589; *P=*0.044). L_RHI did not correlate with HbA1c or fasting plasma glucose levels. Furthermore, L_RHI did not correlate with LDL cholesterol, HDL cholesterol, and triglyceride levels or with systolic and diastolic blood pressures. Finally, multivariate analysis identified MAGE as the only significant determinant of L_RHI.

**Conclusions:**

Fluctuations in blood glucose levels play a significant role in vascular endothelial dysfunction in type 2 diabetes.

**Trial registration:**

UMIN000007581

## Background

Death due to ischemic heart disease and onset of myocardial infarction are approximately 2 to 6 times greater and the risk of stroke is approximately 2 to 3 times greater in patients with type 2 diabetes than normal population [1-5]. Microvascular complications may be ameliorated and/or inhibited by the control of blood glucose levels through maintenance of hemoglobin A1c (HbA1c) below a critical level. However, the outcome of recent large-scale clinical studies suggested that strict glycemic control using HbA1c alone is not sufficient to reduce the risk of macrovascular complications [6,7]; rather, the total number of deaths remained significantly high after intensive therapy [7]. In this regard, severe hypoglycemia has been reported to be an important cause of increased incidence of sudden cardiovascular death [8].

In addition to HbA1c, any glucose-lowering therapy tailored to reduce diabetic angiopathy should also improve postprandial hyperglycemia, which is known to worsen arteriosclerosis, without causing hypoglycemia. In patients with abnormal glucose tolerance, vascular endothelial dysfunction is considered one of the main changes in the initial stage of atherosclerogenesis [9], and is involved in the onset and progression of macroangiopathy [10].

Research using the glucose clamp method demonstrated that accelerated oxidative stress accompanying fluctuations in blood glucose levels worsens endothelial dysfunction more than constant hyperglycemia [11], but there are a few reports that examined the relationship between fluctuations in blood glucose levels and endothelial dysfunction using instruments commonly available in clinical practice.

The present study was designed to determine the relationship between fluctuations in blood glucose levels and vascular endothelial function using bedside medical devices in order to identify a suitable index for the prediction of vascular endothelial dysfunction.

## Methods

A cross-sectional study was conducted involving type 2 diabetic patients who were admitted to the University of Occupational and Environmental Health, Japan between April and November 2011 for glycemic control. The following inclusion criteria were applied in this study: (1) age, >20 years; (2) blood glucose level at admission of <300 mg/dl, (3) no diabetic ketosis or non-ketotic hyperosmolar coma, and (4) absence of cardiac arrhythmia. Patients with infectious diseases and/or acute coronary syndrome were also excluded from the study. The study protocol was approved by the ethics committees of the University of Occupational and Environmental Health and the participating medical centers. Informed consent was obtained from all subjects.

### Study design

The study protocol included the use of a continuous glucose monitoring (CGM) system (CGMS® System GoldTM, Medtronic Inc., Minneapolis, MN) set at admission day 0 to measure fluctuations in blood glucose levels for 72 consecutive hours. Analysis was limited to the data obtained from the intermediate 48 h of recording to avoid bias due to insertion and removal of CGM or insufficient stability of the monitoring system. All patients received optimal meals (25 kcal/kg of ideal body weight; 60% carbohydrate, 15–20% protein and 20–25% fat) during CGM. Peripheral vascular endothelial function assessed by peripheral vascular arterial tonometry (PAT) provides non-invasive assessment of vascular health [12], and was measured by EndoPAT2000 (Itamar Medical, Caesarea, Israel). PAT allows measurement of reactive hyperemia index (RHI), a marker of vascular endothelial function, and involves quantification of arterial pulsatile volume at rest and during increased shear stress associated with the release of nitric oxide (NO) [13]. PAT was conducted on admission day 3.

### Continuous glucose monitoring system

The mean blood glucose level, standard deviation (SD), mean amplitude of glycemic excursions (MAGE), mean postprandial glucose excursion (MPPGE), percentage of time <70 mg/dl, percentage of time ≥140 mg/dl, and percentage of time ≥200 mg/dl were measured from the data recorded through continuous glucose monitoring (CGM) using self-monitoring blood glucose (SMBG) device. MAGE, which was proposed by Service et al. [14], represents fluctuations in blood glucose levels over a 24-hour period and was calculated from the daily variations in blood glucose level, which was measured continuously by CGM over a period of 2 days. To assess postprandial glucose excursions from CGM data, MPPGE was calculated as the arithmetic mean of the differences between the postprandial peak glucose values and the corresponding preprandial glucose values for meals. Previous studies indicated that interstitial glucose concentrations measured by CGM correlate with venous blood glucose levels [15]. CGM measurements represent glucose concentrations in the interstitial fluid, but since the introduction of the SMBG technique, the measured value is considered to represent blood glucose level.

### Noninvasive vascular function test

The method used for digital measurement vascular function using PAT has been described in detail previously [16]. Based on the circadian variation in peripheral vascular tone, the PAT investigation was performed in all patients between 7:00 and 8:00 am in a quiet, temperature-controlled room (21–24°C). All subjects were examined after an overnight fast and 30-min rest in supine position. The baseline pulse amplitude was recorded during a period of 5 min prior to the induction of ischemia. The latter was induced by placing the blood pressure cuff on the upper arm, while the opposite arm served as a control. The PAT probes were placed on one finger of each hand. After 5 min, the blood pressure cuff was inflated to 60 mmHg above the systolic pressure or 200 mmHg for 5 min and then deflated to induce reactive hyperemia. As a measure of reactive hyperemia, RHI was calculated as the ratio of the average amplitude of the PAT signal over 1 min beginning 1.5 min after cuff deflation (control arm, A; occluded arm, C) divided by the average amplitude of the PAT signal over the 2.5-min time period before cuff inflation (baseline) (control arm, B; occluded arm, D). Thus, RHI = (C/D)/(A/B) × baseline correction. Because RHI has a heteroscedastic error structure, we used a natural logarithm transformation in all analyses.

### Measurement of serum lipids, blood HbA1c, and plasma glucose

Venous blood samples were taken in the morning following an overnight fast. Serum low-density lipoprotein (LDL) cholesterol and high-density lipoprotein (HDL) cholesterol concentrations were measured by standard enzymatic methods with the use of a kit from Kyowa Medex (Tokyo, Japan). Triglycerides were measured by standard enzymatic method (Sekisui Medical Co., Tokyo) with a fully automated analyzer (JCA-BM6050; JEOL, Tokyo). The value of HbA1c (%) is estimated as an NGSP equivalent value (%) derived from the JDS value and calculated by the formula HbA1c (%) = HbA1c (JDS) (%) + 0.4% [17]. Fasting plasma glucose levels were measured using a standard enzymatic method. During the study period, no changes were made to any of the medications used by the study participants for the control of diabetes, lipids, and hypertension.

### Statistical analyses

Data were expressed as mean±standard deviation (SD). The Wilcoxon rank sum test was used for comparison of groups. Factors that influence natural logarithmic-scaled RHI (L_RHI) were analyzed using Pearson correlation analysis for normally distributed variables and Spearman rank correlation for variables with skewed distribution. Multivariate stepwise regression analysis was conducted using L_RHI as the dependent variable and several parameters found to be significantly related to L_RHI on univariate analysis. The level of significance was determined as P<0.05. All statistical analyses were conducted using The Statistical Package for Social Sciences version 16.0 (SPSS Inc., Chicago, IL, USA). The association between glucose variability and vascular function in a sample size of 57 subjects provided power of 85% at a significance level of 0.05 (r = 0.4).

## Results

### Clinical characteristics

Patient characteristics are shown in Table 1. The 57 participants were 64.8±12.1 years old (range: 33–87 years), with a disease duration of 14.0±11.9 years, fasting plasma glucose level of 161.3±46.5 mg/dl, and HbA1c of 9.0±1.6% (range: 6.3–15.4%). The study group included slightly higher number of men, who were slightly overweight. Forty-eight patients were treated with oral glucose-lowering agents or insulin. Eleven (19.3%) patients were diagnosed with various cardiovascular diseases.

**Table 1:** Patient characteristics

Age (years)	64.8±12.1
Gender (male/female)	32/25
Body mass index (kg/m^2^)	25.6±4.8
Duration of diabetes (years)	14.0±11.9
Diabetes therapy	
diet only	9 (15.8)
sulfonylurea	21 (36.8)
pioglitazone	11 (19.3)
metformin	8 (14.0)
α-glucosidase inhibitor	9 (15.8)
dipeptidyl peptidase-4 inhibitor	15 (26.3)
insulin	15 (26.3)
Other treatments	
Lipid-lowering drugs	32(56.1)
Antihypertensive drugs	31(54.4)
Current smokers	16 (28.1)
Prevalence cardiovascular disease	11 (19.3)
Systolic blood pressure (mmHg)	134.1±20.9
Diastolic blood pressure (mmHg)	80.3±13.4
LDL-cholesterol (mg/dl)	118.2±35.7
HDL-cholesterol (mg/dl)	51.7±17.2
Triglyceride (mg/dl)	136.2±90.5
HbA1c (%)	9.0±1.6
Glucose	
Fasting plasma glucose (mg/dl)	161.3±46.5
Average (mg/dl)^a^	176.5±47.8
SD (mg/dl) ^a^	42.3±13.2
MAGE (mg/dl) ^a^	110.3±33.4
MPPGE (mg/dl)^a^	79.9±33.7
Time at <70 mg/dl (%)^a^	1.4±3.4
Time at ≥140 mg/dl (%)^a^	67.2±28.4
Time at ≥200 mg/dl (%)^a^	31.4±29.3
L_RHI	0.5±0.2
Baseline pulse amplitude	
Total	8.1±3.5
Men	8.1±3.1
Women	8.0±3.9

Data are mean±SD, n, or n (%). ^a^ Measured by the continuous glucose monitoring system.

Abbreviations: *LDL* low-density lipoprotein; *HDL* high-density lipoprotein; *HbA1c* hemoglobin A1c; *SD* standard deviation; *MAGE* mean amplitude of glycemic excursion; *MPPGE* mean postprandial glucose excursions; *L_RHI* the natural logarithmic scaled reactive hyperemia index.

CGM in the 57 patients showed a mean glucose level of 176.5±47.8 mg/dl (range: 108–321 mg/dl) during the assessment period. The proportion of time spent in hypoglycemia during the same period was 1.4±3.4% (range: 0–16%). The proportion of time spent at glucose level ≥ 140 mg/dl was 67.2±28.4% (range: 12–100%), and that at glucose level ≥ 200 mg/dl was 31.4±29.3% (range: 0–100%). SD was 42.3±13.2 mg/dl (range: 18–73 mg/dl), MAGE was 110.3±33.4 mg/dl (range: 37–193 mg/dl), MPPGE was 79.9±33.7 mg/dl (range: 21.7-192.7 mg/dl), the mean L_RHI was 0.5±0.2 (men: 0.5, women: 0.5) (range: 0.1–1.1) and the baseline pulse amplitude was 8.1±3.5 (men: 8.1, women: 8.0) (range: 0.7-15.3).

### Relationship between L_RHI and markers of diabetic control and nonglycemic metabolic variables

Table 2 lists the correlation coefficients of the relationships between L_RHI and markers of diabetic control and nonglycemic metabolic variables. The univariate analysis showed that L_RHI was significantly related with SD (r=−0.504; *P*<0.001), MAGE (r=−0.571; *P*<0.001), MPPGE (r=−0.411; *P*=0.001) and the percentage of time spent with glucose ≥200 mg/dl (r=−0.292; *P*=0.028). The correlation between L_RHI and percentage of time at hypoglycemia was significant in 12 hypoglycemic patients (r = −0.589; *P* = 0.044) but not in all patients (r = −0.174; *P* = 0.195). On the other hand, L_RHI did not correlate with HbA1c or mean glucose level. Finally, L_RHI did not correlate with the levels of LDL cholesterol, HDL cholesterol, and triglycerides, or with systolic and diastolic blood pressures.

**Table 2:** Correlation coefficients between L_RHI and markers of diabetic control and various nonglycemic metabolic variables

	**HbA1c**	**Average**^**a**^	**SD**^**a**^	**MAGE**^**a**^	**MPPGE**^**a**^	**Time at**			**LDL-C**	**HDL-C**	**TG**	**SBP**	**DBP**
						**<70**^**a **^**(n=12)**	**≥140**^**a**^	**≥200**^**a**^					
Average^a^ (n=57)	0.14												
SD^a^ (n=57)	−0.16	0.36**											
MAGE^a^ (n=57)	−0.06	0.30*	0.79**										
MMPGE^a^ (n=57)	0.01	0.37**	0.64**	0.58**									
Time at <70^a^ with hypoglycemia (n=12)	0.29	−0.69**	−0.38	−0.70*	−0.19								
Time at ≥140^a^ (n=57)	0.12	0.84**	0.27*	0.18	0.32*	−0.68^*^							
Time at ≥200^a^ (n=57)	0.14	0.95**	0.43**	0.37**	0.39*	−0.70*	0.76**						
LDL-C (n=57)	0.26	−0.01	−0.23	−0.13	0.04	0.65*	−0.10	−0.04					
HDL-C (n=57)	−0.17	0.01	0.31*	0.04	0.19	0.03	0.17	0.05	0.06				
TG (n=57)	0.27*	0.05	−0.21	−0.03	0.06	−0.33	0.04	0.01	0.16	−0.28*			
SBP (n=57)	−0.07	−0.25	−0.09	0.01	−0.03	−0.07	−0.19	−0.22	−0.07	−0.18	0.05		
DBP (n=57)	0.20	−0.20	−0.27*	−0.12	−0.10	−0.07	−0.16	−0.22	0.09	−0.26*	0.17	0.65**	
L_RHI (n=57)	0.12	−0.24	−0.50**	−0.57**	−0.41**	−0.59*	−0.20	−0.29*	0.17	−0.14	0.01	0.11	0.09

^a^ Measured by the continuous glucose monitoring system.

Data are results of Pearson correlation analysis for normally distributed variables and Spearman rank correlation for variables with skewed distribution. *P<0.05, **P<0.01.

Abbreviations: *HbA1c* hemoglobin A1c; *SD* standard deviation; MAGE, mean amplitude of glycemic excursions; *MPPGE* mean postprandial glucose excursions; *LDL-C* low-density lipoprotein cholesterol; *HDL-C* high-density lipoprotein cholesterol; *TG* triglyceride; *SBP* systolic blood pressure; *DBP* diastolic blood pressure; *L_RHI* the natural logarithmic scaled reactive hyperemia index.

Multivariate analysis with L_RHI as the dependent variable, and age, gender, body mass index (BMI), duration of the disease, Hypoglycemic agents, Antihypertensive drugs, Lipid-lowering drugs, Prevalence cardiovascular disease, LDL cholesterol, HDL cholesterol, triglycerides, systolic blood pressure, SD, MAGE, the percentage of time with blood glucose <70 mg/dl, and the percentage of time with blood glucose ≥200 mg/dl as the independent variables, identified MAGE (Adjusted multiple R^2^=0.314, Standardized coefficient β=−0.571, t=−5.162, *P*<0.001) as the only independent and significant determinants of L_RHI (Table 3).

**Table 3:** Linear multivariate analyses with L_RHI as the dependent variable.

**Variables**	**Unstandardized coefficients**		**Standardized coefficients β**	**t**	**P value**
	**B**	**SE**			
Intercept	0.923	0.083		11.088	<0.001
MAGE	−0.004	0.001	−0.571	−5.162	<0.001
Adjusted Multiple R^2^	0.314				

Multivariate stepwise regression analysis with L_RHI as the dependent variable and age, gender, BMI, duration of the disease, Hypoglycemic agents, Antihypertensive drugs, Lipid-lowering drugs, Prevalence cardiovascular disease, LDL-cholesterol, HDL-cholesterol, triglyceride, systolic blood pressure, HbA1c, SD, MAGE, Time at blood glucose <70 mg/dl and Time at blood glucose ≥200 mg/dl as the independent variables.

MAGE, Time at blood glucose <70 mg/dl and Time at blood glucose ≥200 mg/dl were measured by the continuous glucose monitoring system.

Abbreviations: *SE* Standard error; *LDL* low-density lipoprotein; *HDL* high-density lipoprotein; *HbA1c* hemoglobin A1c; *SD* standard deviation; *MAGE* mean amplitude of glycemic excursions; *L_RHI* the natural logarithmic scaled reactive hyperemia index.

Rubinshtein et al. [18] reported that cardiovascular events were significantly higher in patients with L_RHI values of <0.4. Accordingly, we divided the patients into two groups, the low L_RHI group (L_RHI <0.4, n=16) and the high L_RHI group (L_RHI ≥0.4, n=41), and investigated glucose fluctuations in the two groups. MAGE was 103.2±30.5 mg/dl in the high L_RHI group, and significantly lower than in the low L_RHI group (128.8±34.4 mg/dl, *P*=0.014). Furthermore, the mean glucose level was 187.6±48.4 mg/dl in the low L_RHI group and 172.2±47.5 mg/dl in the high L_RHI group, but these two values were not significantly different (*P*=0.143).

## Discussion

Previous studies reported that RHI correlates with vascular endothelial dysfunction and is a risk factor for the onset of cardiovascular events [12,19,20]. Based on this background, we used RHI as an index of vascular endothelial dysfunction. This study clarifies the relationship between vascular endothelial function and glucose metabolism, in particular, fluctuations in blood glucose levels.

While there was no relationship between L_RHI and indexes of mean hyperglycemia such as HbA1c and/or mean blood glucose level, vascular endothelial dysfunction correlated with SD, MAGE, MPPGE and the percentage of time spent with glucose ≥200 mg/dl, a measure of fluctuation of blood glucose level and known to denote acute hyperglycemia, as well as with the percentage of time at hypoglycemia. Furthermore, multivariate analysis identified MAGE as the only determinant of vascular endothelial dysfunction in this study. Vascular endothelial dysfunction occurs in the early stage of atherosclerogenesis and is involved in the progression of atherosclerosis [9]. Buscemi et al. [21] reported that glycemic variability influenced endothelial function even in non-diabetic subjects. Monnier et al. [22] indicated that large fluctuations in postprandial blood glucose levels measured by CGM increased oxidative stress more than high blood glucose levels with HbA1c as an index. Using the glucose clamp method, Ceriello et al. [11] reported that worsening of oxidative stress in association with blood glucose fluctuations resulted in endothelial deterioration and dysfunction that exceeded the harmful effects of constantly high blood glucose levels. Recently, Rizzo et al. [23] reported that MAGE reduction is associated with reduction of oxidative stress and markers of systemic inflammation by treatment of dipeptidyl peptidase-IV inhibitor in type 2 diabetic patients. Monnier et al. [24] reported that any strategy should target markers of glycemic variability such as MAGE to limit the risk of complications. The present study examined the relationship between blood glucose fluctuations and endothelial dysfunction using instruments commonly available in clinical practice, i.e., CGM and PAT.

In cases with normal glucose tolerance, vascular endothelial function correlates with postprandial glucose fluctuation, insulin resistance, and postprandial serum insulin level [25], and within the normoglycemic range, individuals whose 2-h plasma glucose levels did not return to their fasting plasma glucose levels during OGTT had elevated fasting insulin levels and increased incidence of coronary heart disease [26]. Patients with mild abnormalities in glucose metabolism are reported to suffer from endothelial dysfunction. Transient hyperglycemia induces oxidative stress [27], the release of proinflammatory cytokines [28], increases reactive oxygen species (ROS) [29], and reduces NO production in association with increased levels of asymmetric dimethylarginine (ADMA) [30]. These reactions are mediated through the translocation and activation of nuclear factor-κB (NF-κB) and the products are thought to be involved in vascular endothelial dysfunction. Su et al. [31] reported that glucose excursion and hs-CRP are associated with the presence and severity of CAD and that glycemic variability measured by CGM correlates with interleukin-6 (IL-6) concentration [32]. The present study also demonstrated that vascular endothelial dysfunction also correlated with MPPGE and the time spent at blood glucose level of ≥200 mg/dl, in addition to postprandial hyperglycemia. In agreement with Di Flaviani et al. [33], established postprandial hyperglycemia is a detrimental factor in reduced flow-mediated vasodilation and activation of oxidative stress is associated with increased glucose variability in type 2 diabetes. In contrast, Peña et al. [34] reported that glucose variability, as measured by CGM, is not associated with vascular function in type 1 diabetes. This highlights possible differences in the role of glucose variability in arteriosclerosis in type 1 and type 2 diabetes.

Although the sample size was very small, the results of the present study highlighted the role of hypoglycemia in vascular endothelial dysfunction. There was no significant difference in the reduction of risk for macrovascular complications between the standard treatment and consolidation treatment groups in the ACCORD study; rather, the total number of deaths was significantly higher [7], and the number of sudden cardiovascular deaths was also higher [8] in patients with severe hypoglycemia. What are the underlying mechanisms of the poor outcome in patients with hypoglycemia? While the present study did not investigate this important question, others have reported the potential roles of sympathetic nerve activation, proinflammatory cytokine secretion, and activation of coagulation factors in hypoglycemic-induced cardiovascular events [35].

In a large community-based cohort, the factors associated with L_RHI were male sex, diabetes mellitus, use of anti-hypertension drugs and the ratio of total cholesterol to HDL cholesterol [36]. However, in that study, L_RHI values did not correlate with male sex, hypertension drugs or markers of cholesterol metabolism. The difference in L_RHI between men and women was related to sex-specific determinants of endothelial function or, alternatively, to the presence of higher baseline pulse amplitude in men compared to women [36]. In the present study, there was no difference in baseline pulse amplitude between males and females, which may explain why there was no difference in L_RHI values.

The present study has certain limitations. First, the subjects were patients admitted to the hospital. The mean blood glucose level measured by CGM was 176.5 mg/dl (9.8 mmol/l). However, the mean HbA1c of the patients was 9.0%; thus, the calculated mean blood glucose level corresponding to this HbA1c value is 234.6 mg/dl (13.0 mmol/l) [37]. This discrepancy was possibly related to the strict diet provided during hospitalization. Second, the results showed no correlation between L_RHI and a marker of fasting cholesterol metabolism. This finding was probably related to the small sample size and treatment of approximately half of the patients with statin. Third, this study was cross-sectional, which precluded evaluation of the cause–effect relationship between glycemic variability and vascular function. Fourth, we could not assess the relationship between oxidative stress or inflammation and PAT. Fifth, the sample size in the present study was relatively small; therefore, subgroup comparisons may lack statistical power. Finally, because the number of subjects in the present study was small, our results should be verified in another study that includes a larger number of subjects.

## Conclusions

In conclusion, the present study demonstrated that vascular endothelial function is influenced by fluctuations in blood glucose levels and acute hyperglycemia of >200 mg/dl. Further well-designed studies of patients with type 2 diabetes are necessary to investigate the long-term effects of fluctuations in blood glucose levels on endothelial function and the possible contribution of such dysfunction on the development of diabetic angiopathies.

## Abbreviations

CGM: Continuous glucose monitoring; RHI: Reactive hyperemia index; L_RHI: Natural logarithmic-scaled reactive hyperemia index; MAGE: Mean amplitude of glycemic excursions; MPPGE: Mean postprandial glucose excursions; HbA1c: Hemoglobin A1c; PAT: Peripheral vascular arterial tonometry; NO: Nitric oxide; SMBG: Self-monitoring blood glucose; LDL: Low-density lipoprotein; HDL: High-density lipoprotein; SD: Standard deviation; BMI: Body mass index; ROS: Reactive oxygen species; ADMA: Asymmetric dimethylarginine; NF-κB: Nuclear factor-κB; hs-CRP: High-sensitivity C-reactive protein; IL-6: Interleukin-6.

## Competing interests

Dr. Tanaka received consulting fees, lecture fees, and/or honoraria from Mitsubishi-Tanabe Pharma, Chugai Pharma, Eisai Pharma, Pfizer, Abbott Immunology Pharma, Daiichi-Sankyo, Janssen Pharma, Astra-Zeneca, Takeda Industrial Pharma, Astellas Pharma, Asahi-kasei Pharma and GlaxoSmithKline, and research grants from Mitsubishi-Tanabe Pharma, Bristol-Myers Squibb, Takeda Industrial Pharma, MSD, Astellas Pharma, Eisai Pharma, and Chugai Pharma.

## Authors’ contributions

All authors listed on the manuscript participated in the design of the study and in writing the manuscript. KT and HM performed the statistical analysis. All authors read and approved the final manuscript.
